# A Comparative Study on Closed Reduction vs. Open Reduction Techniques in the Surgical Treatment of Rotated Lateral Condyle Fractures of the Distal Humerus in Children

**DOI:** 10.3389/fped.2022.891840

**Published:** 2022-06-02

**Authors:** Liuqi Weng, Yujiang Cao, Ge Zhang, Hai Zhou, Xing Liu, Yuan Zhang

**Affiliations:** ^1^Department of Orthopaedics, National Clinical Research Center for Child Health and Disorders, Ministry of Education Key Laboratory of Child Development and Disorders, Children's Hospital of Chongqing Medical University, Chongqing, China; ^2^Chongqing Key Laboratory of Pediatrics, Chongqing, China

**Keywords:** lateral condyle fractures of humerus, humerus, CRPP, ORPP, children

## Abstract

**Objective:**

The best approach between closed reduction and open reduction in the treatment of total displaced and rotated LCFs is still being debated. This study aimed to comparatively evaluate the clinical outcomes and complications of closed reduction vs. open reduction in the treatment of displaced and rotated lateral condyle fractures in children.

**Methods:**

We retrospectively evaluated 46 children who underwent surgical treatment for totally displaced and rotated lateral condyle fractures. Thirty-one children underwent open reduction and percutaneous pinning (ORPP). Ten children underwent closed reduction and percutaneous pinning (CRPP). Five children were changed to ORPP procedures because of the failure of closed reduction attempts. Clinical outcomes and complications in the groups were compared.

**Results:**

Among three groups, no significant differences were found in demographic variables, and no differences were detected in the incidence of postoperative complications and clinical parameters. The ORPP group had the shortest surgical duration of the three groups (*p* < 0.005). Patients in CRPP group had faster fracture healing than the patients who underwent open reduction procedures. However, the success of CRPP seemed to be dependent on the earlier surgical intervention.

**Conclusion:**

ORPP is still the first-line treatment for the totally displaced and rotated lateral condyle fractures because of its direct visualization of the joint surface and easy-to-accomplish characteristics. In addition, CRPP may be a feasible option for the treatment of this type of fractures because of it is less invasive and potentially minimizes complications. However, the technical difficulties of CRPP must be taken into account.

## Introduction

Lateral condyle fractures (LCF) of the distal humerus are the second most common fracture above the elbow in children and commonly occur between ages 5 and 10 years (1). The incidence of LCF has been reported as 12% to 20% of all pediatric upper extremity fractures (2). The most common reported mechanism of injury is avulsion from a fall onto the outstretched arm with a varus stress at the elbow (3). Timely and appropriate evaluation and treatment are necessary to prevent some intractable complications such as avascular necrosis, nonunion, stiffness, and deformity of the affected elbow.

The widely accepted treatment algorithm for LCF has been established in previous studies (4, 5). Briefly, fractures with <2 mm of displacement can be treated initially just with immobilization alone; however, careful follow-up is needed to identify further displacement (6). When lateral condyle fractures are displaced more than 2 mm, operative treatment is recommended (5–7). Open reduction and fixation with Kirschner wires or screws has been used for the treatment of displaced LCF for many decades. With the direct visualization of the articular surface, an anatomic reduction can be achieved for this kind of intra-articular fracture (8). However, some recent studies reported satisfactory outcomes of closed reduction and percutaneous pinning (CRPP) in treating displaced lateral condyle humeral fractures (9). This technique is most commonly used for displaced fractures with an intact cartilage hinge or no notable fragment malrotation (10). Generally, CRPP has been advocated for children with LCF displaced more than 2 mm but <4 mm and without obvious articular surface incongruity under intraoperative arthrography. Otherwise, if the LCF is displaced more than 4 mm with or without fragment rotation, open reduction and percutaneous pinning (ORPP) is seen as the most optimal choice (5, 11).

CRPP has shown several advantages over ORPP, including less dissection of soft tissue around the fragment, low risk of vessel damage, and avoidance of an open incision with an unaesthetic scar (12). In recent decades, CRPP has been utilized to deal with displaced and rotated LCF successfully and seems to be an attractive alternative to ORPP. Song et al. (4) reported excellent results in three of six displaced and rotated LCF with the use of CRPP. Their following study reported more encouraging evidence that 18 of the 24 of displaced and rotated LCFs had achieved satisfactory results (13). However, the unavoidable fact is that the learning curve for this technique is time-consuming, and uncertainty over reduction of a substantially displaced LCF is still a concern. Whether closed reduction or open reduction is the best approach in the treatment of total displaced and rotated LCFs is still being debated. The purpose of this study was to comparatively evaluate the outcomes of closed reduction vs. open reduction in treating displaced and rotated LCFs (Stage-5 LCF according to the Song classification) (4) to provide a reference for treatment selection to peers when encountering this type of injury.

## Methods

### Patient Selection

This study was approved by the Institutional Review Board of Children's Hospital of Chongqing Medical University. We retrospectively enrolled consecutive children with displaced LCF surgically treated at our institution from August 2018 to May 2020. The inclusion criteria were (1) patients below 14 years of age, (2) patients diagnosed with displaced and rotated LCF (Stage 5 LCF according to the Song classification), (3) interval from injury to admission <5 days, and (4) more than 6 months' clinical and radiographic follow up. The exclusion criteria were (1) combination with ipsilateral upper-limb fracture and/or dislocation, (2) pathological fracture, and (3) open fracture. In total, 46 patients with displaced LCF were enrolled in this study. Written informed consent was obtained from the parents or guardians of each patient. Closed or open reduction was determined by the consensus reached by the children' guardians and surgeons.

### Surgical Techniques

**Figure 1:**
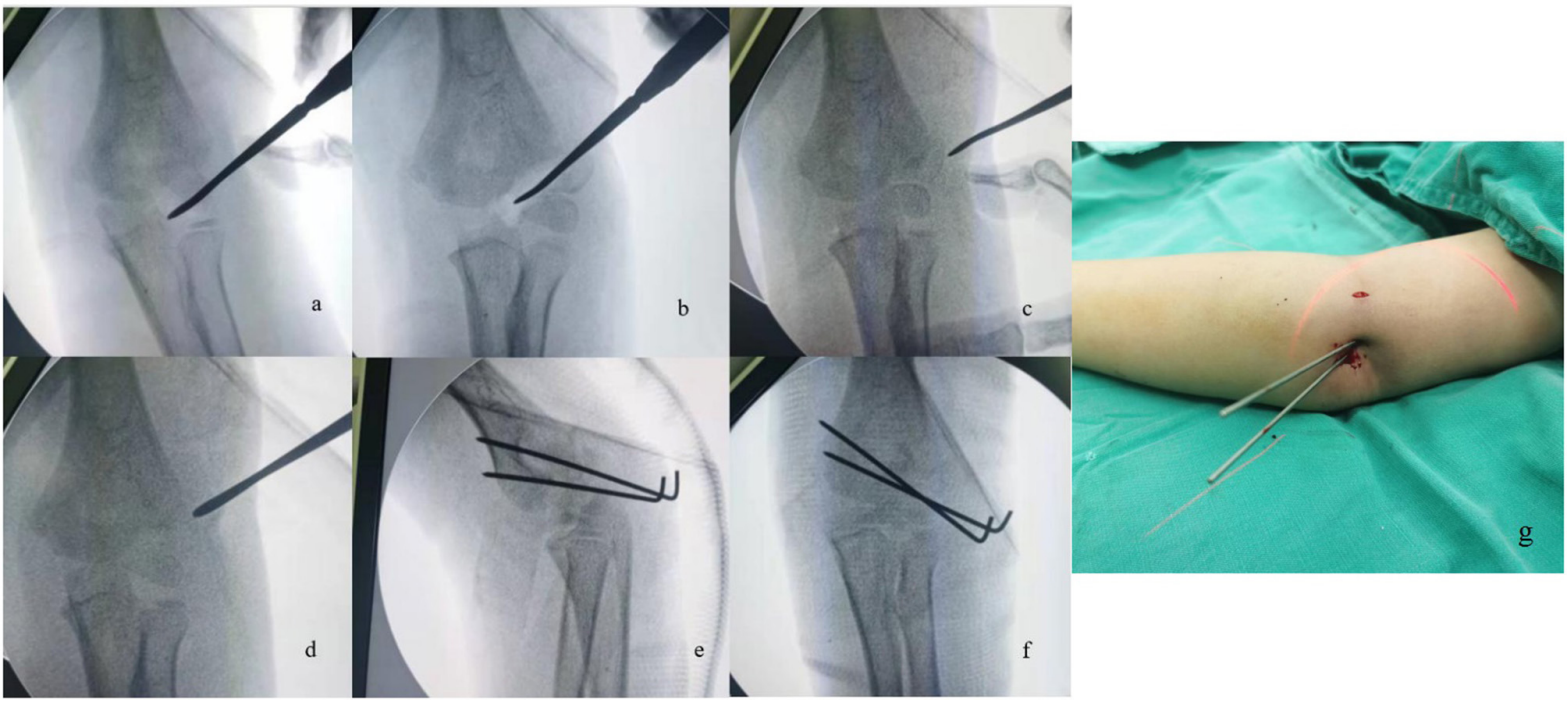
**(a)** A Dura dissector was placed into the lateral cortex under c-arm fluoroscopy. **(b)** The rotated fragment was reduced by Dura dissector prying. **(c)** The elbow was fully extended with forearm supination, and direct compression was applied by a surgeon's thumb on the distal fragment medially and anteriorly to minimize the fracture gap. **(d)** Assurance that the fracture gap was no more than 2 mm. **(e,f)** Two percutaneous k-wires were inserted for fixation in AP and oblique internal rotational view. **(g)** The minimal lateral incision after the CRPP procedure in the treatment of LCFs.

When closed reduction was attempted, the surgical technique reported by Song et al. was employed (4, 13). The procedure was performed under general anesthesia with the children in the supine position. The displacement of the fracture of the affected elbow was reconfirmed under intraoperative fluoroscopy. The rotated displacement of the distal fragment was the first to be reduced. The affected elbow was placed in flexion in an appropriate position to relax the stretching of forearm extensors, usually about 40–60° flexion. Different from the original method described by Song et al. in which a Kirschner wire was used as a joystick to assist reduction, we were accustomed to using a Davis dura dissector as the joystick because its wide tails made it more easily manipulated when reducing the rotated fragment. The Davis dura dissector was inserted into the fracture gap through a minimal lateral elbow incision (about 5 mm in length). Then, an attempt was made to reposition the rotated fragment by using the dissector to pry open the fragment, with a view to make the two fracture surfaces in an opposite position. After the fragment rotation was corrected, the elbow was fully extended with the forearm supine, and direct compression was applied by a surgeon's thumb on the distal fragment medially and anteriorly to minimize the fracture gap. After assurance that the fracture gap was no more than 2 mm either in the AP or oblique internal rotational view, two or three percutaneous K-wires (1.6 or 1.8 mm in diameter) were inserted for fixation. Then an intraoperative arthrogram was used to confirm the congruence of the articular surface of the distal humerus (Figure 1). For fractures with > 2 mm of displacement or incongruence of the articular surface following closed reduction, an open reduction was employed. The ORPP technique was undertaken as described by Blasier (14). The patient was placed in the supine position and a tourniquet was utilized. A direct lateral incision was made and care was taken to minimize posterior dissection of the capitellum. Under direct visualization, the articular surface was reduced and stabilized with two to three divergent K-wires with diameters of 1.6 or 1.8 mm that engaged the medial cortex. Thereafter, the affected arm was placed in a posterior long-arm cast with a 45° of elbow flexion to immobilize the fracture about 4–6 weeks until the removal of the K-wires. The K-wires were removed in the outpatient clinic when fracture healing was documented on two views. All children had at least six months of follow up and complications were noted.

### Clinical Outcomes Evaluation

At the last follow up, the range of motion (ROM) of the elbows and elbow carrying angle were evaluated using a goniometer. The loss of ROM and elbow carrying angle were defined by the difference in values between the affected side and contralateral normal side. In addition, the functional and cosmetic outcomes of the affected elbow were assessed according to Flynn's criteria (15). The occurrences of clinical complications such as infections (superficial/deep), late ulnar neuritis, and conspicuous incision scar after surgery were also recorded. More specifically, the superficial infection was defined as the infection involving only skin and subcutaneous tissue of incision, with little or no tissue reaction. The deep infection involved deep tissues, such as fascial and muscle layers, even at the fracture site.

### Radiographic Outcomes Evaluation

The radiographic outcomes were evaluated in AP and lateral radiographs of elbows at each follow up in all the patients. Osseous union was confirmed by the presence of bone bridging on AP and lateral radiographs. Cases with delayed union, nonunion, and malunion were recorded. Furthermore, avascular necrosis of the humeral capitulum, fishtail deformity at distal humerus, and lateral spur formation were also assessed from the postoperative radiographs. The radiographic carrying angle were measured on the AP radiographs.

### Statistical Analysis

All variables were analyzed by the SPSS 22.0 statistical software, continuous data were indicated by mean ± SD, and the ANOVA analysis and independent sample *t*-test were used for the comparison of continuous variables. The chi-square test was used for categorical variables. The Kruskal-Wallis test was used for ranked variables. The level of statistical significance was set at *p* < 0.05.

## Results

A total of 46 patients who met the inclusion criteria underwent surgical treatment for the diagnosis of a displaced and rotated LCF from August 2018 to May 2021. There were 26 males (56.5%) and 20 females (43.5%) included in this study. Thirty-one fractures were directly treated with ORPP and all the fractures achieved successful reduction. In April 2020, we began to use CRPP to treat LCF with complete displacement and rotation of fragments. To sum up, treatment of 15 fractures was initially attempted with CRPP. Of these, 10 (10/15, 66.7%) fractures were successfully treated with CRPP, but the other 5 fractures (5/15, 33.3%) needed to be changed to the ORPP procedure because the fracture gap was more than 2 mm or there was incongruence of the articular surface on the arthrogram after closed reduction efforts. In addition, the patients who were converted to open reduction were the first, second, fifth, sixth, and tenth patients in the CRPP cohort. All the conversions occurred in the first 10 patients. A summary of variables compared by treatment types is shown in Table 1. No differences were found among any of the groups in age, sex, follow-up duration, and side of injury. The interval from injury to surgery, surgery duration, and fracture healing time had differences among groups. A shorter interval time from injury to surgery was found in than CRPP group than in the ORPP (*p* < 0.001) or converted groups (*p* < 0.001). The ORPP group had the least time, 36.00 ± 9.16 min, for surgical completion, and the converted group had the longest time, 81 ± 8.43 min, to finish the surgery. The mean fracture healing time in the CRPP group was 4.50 ± 0.53 wk, which was shorter than those in the other two groups.

**Table 1:** General descriptive data of three groups.

**Variables**	**Treatment**						
	**ORPP**	**CRPP**	**Converted group**	**P**	**^**#**^P**	**^**&**^P**	**^**%**^P**
No. of children	31	10	5				
Age at the presentation (years)	5.39 ± 2.03	4.90 ± 2.33	5.00 ± 2.00	0.782			
Sex							
Male	17	6	3	>0.999			
Female	14	4	2				
Side of injury							
Left	16	6	3	0.92			
Right	15	4	2				
Neurovascular involvement	0	0	0				
Interval from injury to surgery (days)	3.23 ± 0.72	2.50 ± 0.53	3.60 ± 0.55	0.005^*^	<0.001^*^	0.275	0.002^*^
Surgery duration (minutes)	36.00 ± 9.16	56.1 ± 9.99	81 ± 8.43	<0.001^*^	<0.001^*^	<0.001^*^	<0.001^*^
Fracture healing (weeks)	5.84 ± 1.34	4.50 ± 0.53	6.20 ± 0.84	0.007^*^	0.004^*^	0.566	<0.001^*^
Follow up (months)	9.81 ± 3.53	10.20 ± 4.83	10.40 ± 2.70	0.923			

*ORPP, open reduction and percutaneous pinning; CRPP, closed reduction and percutaneous pinning; Converted group, CRPP converted to ORPP; P, statistical significance among three groups*;

# *P: ORPP vs. CRPP*;

& *P: ORPP vs. Converted group*;

% *P: CRPP vs. Converted group*;

* *statistical significance and P-value was less than 0.05*.

Data were collected on complications including infections (superficial/deep), delayed union, nonunion, malunion, late ulnar neuritis, lateral spur formation, avascular necrosis, fishtail deformity, and conspicuous incision scar after surgery. No differences in these variables were found among the three groups (Table 2).

**Table 2:** Complications in three groups.

**Complications**	**ORPP**	**CRPP**	**CRPP converted to ORPP**	***p***
Superficial infection	4/31	0/10	1/5	0.725
Deep infection	2/31	0/10	0/5	>0.999
Delayed union	0/31	0/10	0/5	
Nonunion	0/31	0/10	0/5	
Malunion	0/31	0/10	0/5	
Tardy ulnar neuritis	0/31	0/10	0/5	
Lateral spur formation	23/31	7/10	3/5	0.783
Avascular necrosis	0/31	0/10	0/5	
Fishtail deformity	0/31	0/10	0/5	
Conspicuous incision scar	5/31	0/10	0/5	0.459

At the last follow up, the loss of ROM and radiographic elbow carrying angle were defined by the difference in values between the affected side and contralateral normal side. Regardless of the treatment methods, all the injured elbows had a slight decrease either in extension, flexion, and movement arc when compared to the normal contralateral elbow. However, no differences were found among the three groups in these parameters. Moreover, no differences were observed among the three groups in the radiographic carrying angle. In addition, the clinical outcomes were classified as excellent, good, fair, or poor according to Flynn's criteria (15). No significant differences were observed among groups in the cosmetic outcome and functional outcome according to Flynn's criteria (Table 3).

**Table 3:** Radiographic and clinical outcomes of three groups.

	**Treatment**			
	**ORPP**	**CRPP**	**CRPP converted to ORPP**	***p***
Radiographic carrying angle (°)				
Affected side	8.58 ± 5.19	8.90 ± 4.56	5.20 ± 1.64	0.325
Contralateral side	9.03 ± 3.34	10.00 ± 3.27	8.4 ± 1.14	0.601
Loss of carrying angle	0.46 ± 3.35	1.10 ± 3.21	3.20 ± 2.59	0.221
ROM of elbow (extension, flexion, arc)				
Extension (°)				
Affected side	1.45 ± 1.65	1.50 ± 1.08	1.60 ± 0.89	0.978
Contralateral side	3.16 ±1.68	4.30 ± 1.16	4.20 ± 0.84	0.08
Loss of extension	1.71 ± 1.74	2.80 ± 1.14	2.60 ± 1.14	0.129
Flexion (°)				
Affected side	131.29 ± 7.05	126.00 ± 4.81	128.20 ± 5.63	0.081
Contralateral side	136.16 ± 6.48	131.70 ± 4.81	133.40 ± 5.94	0.126
Loss of flexion	4.87 ± 2.51	5.70 ± 2.00	5.20 ± 1.10	0.615
Arc (°)				
Affected side	132.74 ± 7.53	127.50 ± 4.84	129.8 ± 5.76	0.111
Contralateral side	139.32 ± 6.99	136.00 ± 4.19	137.60 ± 6.07	0.356
Loss of flexion	6.58 ± 2.94	8.50 ± 2.12	7.80 ± 2.17	0.141
Flynn's criteria (cosmetic, functional)				
Cosmetic outcome				
Excellent	24	8	3	0.668
Good	7	2	2	
Fair	0	0	0	
Poor	0	0	0	
Incidence of “excellent” or “good”	100%	100%	100%	
Functional outcome				
Excellent	7	0	0	0.206
Good	19	7	4	
Fair	5	3	1	
Poor	0	0	0	
Incidence of “excellent” or “good”	83.90%	70%	80%	

## Discussion

The foremost goal of treatment for LCF in children is to restore the anatomical articular surface. For this reason, open reduction and Kirschner wire fixation has long been considered the preferred method for LCFs. Most of the published studies addressing surgical treatment of LCFs have focused on techniques utilizing an open approach. According to the displacement and congruity of the articular surface of the LCF fractures under arthrography, a classification was proposed by Weiss et al. (5) to guide treatment decision making. Type I fractures are fractures with <2 mm displacement that can be managed with observation and casting. Type II fractures are displaced more than 2 mm but with congruence of the articular surface, which can be managed with closed reduction. Type III fractures have articular surface displacement and open reduction is recommended, although most pediatric orthopedic surgeons do not recommend closed reduction for the treatment of the displaced and rotated lateral condyle fractures (6). This technique for displaced LCFs has received increasing attention. In the last decade, CRPP has still been favored by other surgeons when joint congruity can be confirmed because it is less invasive and potentially minimizes complications, and some promising results have been found (16). The present study also found that the LCFs treated by CRPP had a shorter fracture healing time than those treated by ORPP.

Song et al. conducted a prospective study of CRPP for treating unstable lateral condyle fractures, and achieved a high success rate (73%). However, only three of six (50%) with displaced and rotated lateral condyle fractures were reduced to <2 mm of residual displacement and needed a further open reduction procedure (4). Silva et al. also confirmed that CRPP is a safe and effective alternative when considering the treatment of pediatric LCFs with limited displacement (between 2 and 4 mm) (17). However, after accumulating experience, Song and colleagues achieved a tremendous success rate of 85.7% (18/21) in such fractures using CRPP (13). More recently, a study by Xie et al. (12) demonstrated that CRPP is an effective technique for treating LCFs with severe displacement. The overall success rate of closed reduction was 78% (36/46) regardless of the displacement grade. In addition, 14 of 18 (78%) with displaced and rotated LCFs were also successfully treated with CRPP. In the present study, we used CRPP for 15 lateral condyle fractures with complete displacement and rotation. Only 10 of the 15 (66.67%) fractures were satisfactorily reduced, as defined by the fracture gap being <2 mm either in anteroposterior (AP), lateral, and oblique radiographic views, and the congruent articular surface was confirmed by intraoperative arthrography. This success rate of closed reduction was lower than that in previous studies. We considered that the principal reason for our failure in reduction was the high degree of displacement of fractures in the present study. All the LCFs was displaced and rotated, which indicated that more soft tissue around the fracture fragment had been destroyed. In particular, the massive disruption of the lateral periosteum in this type LCF could give rise to the lack of a support point during manual reduction, which made the surgeons convert to open reduction after unsuccessful closed reduction attempts.

CRPP has been widely accepted as the standard treatment for unstable supracondylar fractures. It was reported that delaying surgery more than 8 h was associated with an increased rate of open reduction (18). The present study focused on CRPP for the treatment of LCFs and also found that increased time from injury to surgery led to a trend toward open reduction. The failure can be ascribed, at least in part, to the significant swelling from delayed treatment making the fracture fragments hard to palpate (19). Moreover, coagulated blood clots between the fracture gap and contracted soft tissues might also have hampered the reduction.

Undoubtedly, accumulated experience is necessary for the skilled manipulation during closed reduction (16). For the same reason, we had a limited success rate (66.67%) for rotated LCFs with CRPP in this study. Among patients who underwent closed reduction attempts, 5 of the 15 patients underwent conversion to open reduction, 3 of them occurred in the first five cases (60%), and all of them occurred in the first 10 patients. The time-consuming learning curve of closed reduction for the rotated LCFs cannot be ignored. More experience and training may help us to be more proficient with this technique.

The open reduction procedure allows direct visualization of the joint surface, and for this reason, getting a congruent joint surface and maintaining reduction can be easily guaranteed (20). Closed reduction has been favored by some colleagues because it requires less dissection of soft tissue and avoids incision and a conspicuous scar, with lower risk of complications (4). However, in the present study, using open reduction internal fixation (ORIF) to treat rotational LCFs did not increase the risk of complications when compared to the fractures treated by CRPP. Moreover, comparable satisfactory functional outcomes and cosmetic outcomes have been obtained by both open reduction and closed reduction. Interestingly, unlike the previous reports that CRPP had a shorter operating time than open reduction procedures in treating LCFs, the present study found that the CRPP procedure in treating rotational LCFs takes a significantly longer time than that in ORIF to achieve a satisfactory reduction and fixation. In our experience, technical difficulty might be the main reason for the long duration of CRPP in treating this type of rotational LCFs. In addition, repeated intraoperative confirmation of the reduction and secure maintenance of percutaneous K-wires are also time-consuming processes. According to present outcomes, we still hold the cautious view that the ORPP might be still the first-line treatment for total displaced and rotated LCFs.

Lateral spur formation was the most common complication in the present study, but almost all of them were asymptomatic. Our results were consistent with the previous studies (21). In addition, we observed a comparable incidence of lateral spur formation among the three groups. Regardless of the surgical methods, all the LCFs in present study were fixed with the K-wires, which is not a rigid fixation system. As a result, micromotion between the bone fragments at the fracture site enhanced the bone formation and led to the lateral spur formation.

In conclusion, both CRPP and ORPP in treating total displaced and rotational LCFs yield good clinical outcomes and acceptable complication incidences on the basis of successful reduction and fixation achieved intraoperatively. However, because of the standardization of the operative process and straightforward characteristics, open reduction and fixation remains the “gold standard” for displaced and rotated LCFs, especially for patients with a longer interval between injury to first treatment. Nevertheless, with the intrinsic advantages such as faster healing time without risk of conspicuous incision scar and lower surgical infection rate, CRPP should be still taken into consideration in the decision-making process for the treatment of this type LCF, and surgeons should be prepared for the time-consuming learning process.

## Data Availability Statement

The raw data supporting the conclusions of this article will be made available by the authors, without undue reservation.

## Ethics Statement

This was a retrospective study of patient data, and IRB approval was obtained from Children's Hospital of Chongqing Medical University (2020196). Written informed consent to participate in this study was provided by the participants' legal guardian/next of kin.

## Author Contributions

LW, XL, and YZ were involved in the conception, design of the project, and made the critical revisions. YZ, LW, HZ, and GZ participated the surgery implementation. LW and YC collected and extracted the data. XL, YC, and YZ conducted the analysis and data interpretation. YZ drafted the manuscript. All authors read, provided feedback, and approved the final manuscript.

## Funding

This work was supported by the Projects of Chongqing Science and Technology Committee Foundation (cstc2019jcyj-msxmX0853), Youth Project of National Clinical Research Center for Child Health and Disorders (NCRCCHD-2021-YP-05), and Chongqing Science and Health Joint Project (2021MSXM303).

## Conflict of Interest

The authors declare that the research was conducted in the absence of any commercial or financial relationships that could be construed as a potential conflict of interest.

## Publisher's Note

All claims expressed in this article are solely those of the authors and do not necessarily represent those of their affiliated organizations, or those of the publisher, the editors and the reviewers. Any product that may be evaluated in this article, or claim that may be made by its manufacturer, is not guaranteed or endorsed by the publisher.
